# Community-Based View on Diagnostic Imaging at the End of COVID-19 Pandemic: Online Survey-Assisted Study

**DOI:** 10.3390/diagnostics14121269

**Published:** 2024-06-15

**Authors:** Nina D. Anfinogenova, Aleksandra S. Maksimova, Tatiana A. Shelkovnikova, Nadezhda I. Ryumshina, Alina D. Kuznetsova, Nazary P. Chesalov, Rostislav S. Karpov, Wladimir Y. Ussov, Alexey N. Repin

**Affiliations:** 1 Cardiology Research Institute, Tomsk National Research Medical Center, Russian Academy of Sciences, 634012 Tomsk, Russia; 2 School of Computer Science & Robotics, National Research Tomsk Polytechnic University, 634050 Tomsk, Russia; alinadk@tpu.ru; 3 Meshalkin National Medical Research Center, 630055 Novosibirsk, Russia

**Keywords:** surveys and questionnaires, diagnostic imaging, magnetic resonance imaging, epidemiology, public health, big data, COVID-19, environmental pollution, rural health services, population health

## Abstract

(1) Background: An online survey-based observational cross-sectional study aimed at elucidating the experience and attitudes of an unstructured population regarding diagnostic imaging. (2) Methods: Invitations to participate were distributed using mixed-mode design to deidentified residents aged 18 years and older. Main outcome measures included morbidity structure and incidence of diagnostic imaging administrations. (3) Results: Respondents (*n* = 1069) aged 44.3 ± 14.4 years; 32.8% suffered from cardiovascular diseases (CVD); 9.5% had chronic respiratory pathology; 28.9% considered themselves healthy. Respondents with COVID-19 history (49.7%) reported higher rates of computed tomography (CT) (*p* < 0.0001), magnetic resonance imaging (MRI) (*p* < 0.001), and ultrasound (*p* < 0.05). COVID-19 history in CVD respondents shifted imaging administrations towards CT and MRI (*p* < 0.05). Every tenth respondent received MRI, CT, and ultrasound on a paid basis; 29.0% could not pay for diagnostic procedures; 13.1% reported unavailable MRI. Professional status significantly affected the pattern of diagnostic modalities (*p* < 0.05). MRI and CT availability differed between respondents in urban and rural areas (*p* < 0.0001). History of technogenic events predisposed responders to overestimate diagnostic value of fluorography (*p* < 0.05). (4) Conclusions: Preparedness to future pandemics requires the development of community-based outreach programs focusing on people’s awareness regarding medical imaging safety and diagnostic value.

## 1. Introduction

The pandemic of 2019-novel-coronavirus infection (COVID-19) dramatically transformed the healthcare system and life of society at every level. Hospitalization and mortality rates in emergency departments doubled in non-COVID and tripled in COVID-19 diagnosis, and these spikes resulted from both patient severity and healthcare system overload [1]. In contrast, admission rates in other tertiary centers significantly decreased due to safety concerns. Remarkable disturbances occurred in delivering diagnostic imaging services at specialized cardiovascular centers during the onset of COVID-19 pandemic, when a significant drop in the number of diagnostic procedures was caused by the restriction measures aimed at providing safety to patients and medical personnel [2,3].

A successful vaccination campaign decreased global burden of acute COVID-19, but large groups of the population continue suffering from persisting symptoms of post-COVID-19 syndrome or long COVID [4,5], while the waves of COVID-19 tend to reoccur [6]. Previous data showed the involvement of the cardiovascular system in the pathogenesis of long COVID, suggesting the need for consistent use of diagnostic algorithms aimed at ruling out the inflammatory changes in the myocardium as it may be lifesaving [2,3,7]. Besides cardiovascular involvement, long COVID manifests with multiple conditions [8], including thrombotic and cerebrovascular abnormalities [9], type 2 diabetes mellitus [10], myalgic encephalomyelitis [11,12], and autonomic failure [12]. Long COVID can last for years [13], becoming a disabling condition in so many people that it may contribute to labor shortages at a national level [8]. Affected individuals require continuous monitoring and preventive medical examinations [2].

Healthcare systems and overall society must take steps for developing viral epidemic preparedness and emergency disaster care plans due to the threat of impending pandemics [1,14,15,16,17,18,19]. The measures should cover all aspects of healthcare, including self-management, clinical laboratory services, diagnostic procedures, outpatient and inpatient care, telemedicine, etc. Future deadly viral pandemics may dramatically increase demand in the early administration of imaging procedures, but the time-consuming nature of their manual delivery by experts [20,21] could significantly limit potentially high flow of affected patients. Moreover, currently available diagnostic procedures involve direct communication between diagnostic specialists and patients, while social distancing may save lives during viral pandemics such as COVID-19 [22,23].

To meet these challenges, researchers over the globe have developed novel approaches such as machine learning, deep neural networks, virtual reality, augmented reality, artificial intelligence (AI), Tactile Internet, and pervasive computing, among others, applied to advanced diagnostic imaging [24,25,26,27,28,29,30]. System architecture has been proposed to integrate the Internet of Things, wireless sensor networks, big data and analytics, cloud computing, machine learning, and 5G networks alongside conventional methods to detect and combat infectious diseases [24]. A novel visualization technique assisted with AI-algorithm-enhanced haptics may offer remote patient examination and treatment through robotics [25]. Chest X-ray image enhancement and segmentation at the preprocessing stage may increase the overall quality and contrast of the images to improve convolutional neural network (CNN)-based recognition of clinically significant patterns [26]. A deep-learning-based automated chest X-ray classification system significantly improves the accuracy of diagnosing respiratory lung diseases, including COVID-19 [27]. Designed automated reporting systems spare time and costs and provide prompt, accurate, and early diagnostic reports with accuracy over 98% during instance segmentation [28]. An explainable deep neural networks (DNN)-based method is promising for automatic detection of COVID-19 symptoms from chest radiography (CXR) images with high positive predictive values for normal, pneumonia, and COVID-19 cases [29]. DNN-based integration of diagnostic imaging and clinical information is used for predicting survival of COVID-19 patients [30].

Medical imaging represents a rapidly evolving valuable resource for combating long COVID and future pandemics, especially if adequately integrated with self-management in cases of healthcare system overload. Implementation of AI-assisted advanced imaging techniques and algorithms would require adequate knowledge, attitudes, and readiness of both medical specialists and medically inexperienced people seeking care. However, while the situation with diagnostic imaging during the last pandemic has been relatively clear from the standpoint of one medical center [2], the experience, attitudes, and expectations of the population in regard to diagnostic services remain poorly understood.

A survey-based approach showed to be promising for identifying the clinically vulnerable population [31] and behavioral determinants of health during the pandemic [32]. The primary objective of our online survey-based study was to gain insights into the real-world data characterizing the experience, awareness, and attitudes of an unstructured population towards their use of diagnostic imaging modalities during and after the COVID-19 pandemic.

The next sections of the paper describe the methodology and survey results regarding diagnostic imaging in the contexts of population age, morbidity structure, diagnostic imaging affordability and availability, respondents’ professional status, perceived superiority of diagnostic imaging modalities, and local factors such as ecological situation and settlement type.

## 2. Materials and Methods

### 2.1. Ethics

The study protocol was approved by the Biomedical Ethics Committee at Cardiology Research Institute of Tomsk National Research Medical Center (Tomsk, Russia) (approval #248 from 27 September 2023). All patients signed the informed consent form online. The present paper is the first of a planned series of research articles reporting data from the study registered at ClinicalTrials.gov ID: NCT06159699.

### 2.2. Depersonalized Online Survey

This study assessed the depersonalized survey questionnaire data of a total of 1069 respondents from several regions of the Russian Federation, including the Amur, Chelyabinsk, Irkutsk, Kemerovo, Leningrad, Magadan, Novosibirsk, Omsk, Rostov, Sverdlovsk, Tomsk, Vladimir, and Volgograd regions; Khabarovsk, Krasnodar, Krasnoyarsk, and Trans-Baikal territories; Primorsky Krai; Jewish Autonomous Province; and Republic of Buryatia and Republic of Tyva. The online questionnaire was available on specialized original websites [33,34] from 1 December 2022 to 31 October 2023.

Only adult respondents aged 18 years and older were invited by text messages sent to deidentified residents by the Target-SMS service provided by the telecommunication company Tele2 Russia. The delivery rate reached 77.4%. The invitees were selected randomly by the Target-SMS software based on the following criteria: (1) compliance of customer to receive target SMS messages; (2) Android or iOS mobile operating systems powering the device; and (3) residence in the selected territories of the country at the time of obtaining the Subscriber Identity Module card. Additionally, the invitations were posted on the Internet (on the VK Social Network and authors’ institution official website).

Mobile and desktop versions of specialized websites [33,34] were developed. The survey questionnaire contained the questions on demographic, social, behavioral, clinical, and environmental factors, as well as people’s experience and attitudes to medical imaging.

No identifiable patient information is provided in the manuscript. This paper contains only aggregate depersonalized data. The participants had the option to provide their contact information (phone number or/and email). Each participant provided signed informed consent online by checking the appropriate box at the website to comply with legal requirements.

### 2.3. Sample Size Calculation

Sufficiency of sample size was established based on the following considerations: the acceptable margin of error of 5%, confidence level of 95%, and approximate population size of 1,000,000. A further increase in the population size estimate did not significantly affect the calculated sample size. Taking into account assumed 50%-response distribution, a sufficient sample size was 385 respondents, calculated as follows: the sample size *n* and margin of error *E* are given by:

(1) x=Z×c1002×r100−r,

(2) n=N×xN−1×E2+x,

(3) E=N−n×xn×N−1,

where *N* represents the population size, *r* is the fraction of responses of interest, and *Z* × (*c*/100) is the critical value for the confidence level *c*. Taking into account that our sample of respondents exceeded the calculated sample size, the sample size was considered sufficient.

### 2.4. Statistical Analysis

Normality of distribution of variables was checked by the Kolmogorov–Smirnov test and the Shapiro–Wilk test. Data are presented as percentages, absolute numbers, probabilities, mean ± standard deviation, and median and interquartile range where appropriate. Correlational analysis was performed by computing Pearson and Spearman’s Rank correlation coefficients where appropriate. Categorical variables were compared by Pearson’s Chi-Square test using multifield contingency tables. Correlation matrix was used to analyze significant associations between the variables. Values were considered statistically significant when *p*-value was < 0.05.

## 3. Results

### 3.1. Sample Characteristics

Distribution of SMS invitations resulted in a total of 3096 persons following the link provided in the text message; 1588 invitees signed the informed consent form and 1069 individuals (67.9%) validly completed the survey questionnaire. The enrolled respondents were aged 44.3 ± 14.4 years and the majority of them were female (73.0%). Age distribution in the cohort was normal in both men and women. Median age did not significantly differ between men and women. Rates of answer choices regarding medical imaging did not significantly depend on sex of respondents. For this reason, the paper presents unadjusted data.

**Figure 1:**
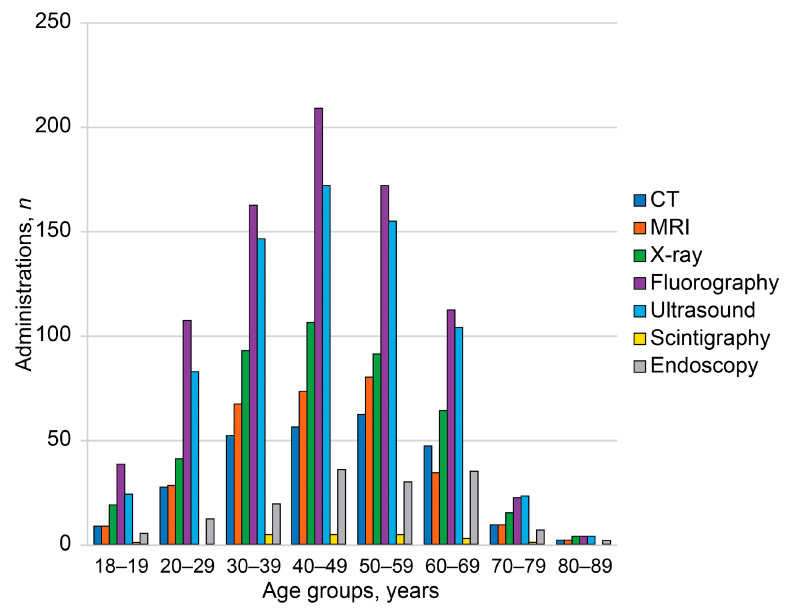
Numbers of administrations of diagnostic imaging modalities in age groups over the entire cohort. The rates were calculated for two years prior to the survey. CT—computed tomography, MRI—magnetic resonance imaging.

**Figure 2:**
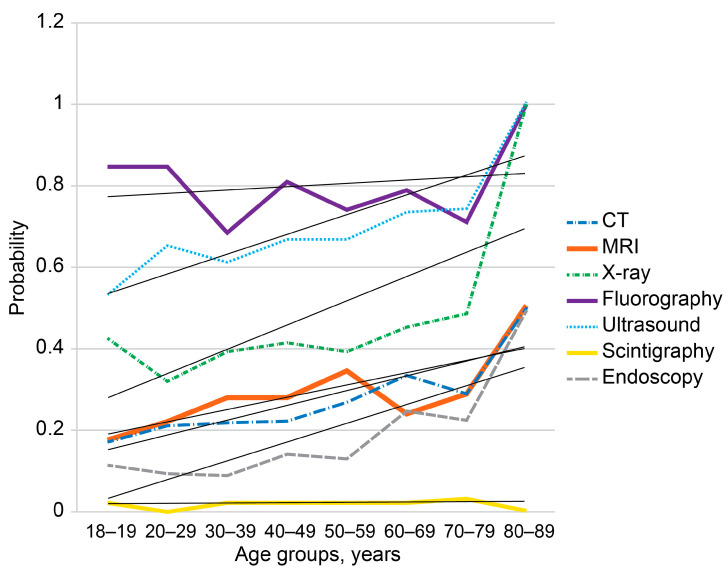
Probabilities of imaging modality administrations per person in age groups. Black lines represent linear trends in age-dependent probabilities. The rates were calculated per person for the past two years prior to the survey. CT—computed tomography, MRI—magnetic resonance imaging.

Respondents reported receiving the following diagnostic imaging examinations within the two years prior to the survey: fluorography (76.7%), ultrasound (66.0%), X-ray (40.2%), MRI (27.9%), CT (24.4%), scintigraphy (1.9%), and endoscopy (13.7%). A total of 6.1% of respondents reported that they did not receive any diagnostic procedures within the two years prior to the survey. Fluorography was the most common imaging modality (Figure 1 and Figure 2).

### 3.2. Age-Related Associations

Cohort-wide numbers of administered imaging modalities were normally distributed, and probabilities of imaging modalities had upward age-related trends (Figure 1 and Figure 2). Significant age-related differences were found between 60–69 year olds and other age groups. The 60–69-year-old group had significantly higher probability of receiving CT compared with 20–49-year-old respondents (*p* < 0.05). However, they had lower probability of MRI compared with 20–59-year-old respondents (*p* < 0.05). The 60–69-year-old group more often received X-ray compared with 20–29-year-olds (*p* < 0.05) and chest fluorography compared with 30–39-year-olds (*p* < 0.05). The probability of receiving ultrasound in this group was higher than in 18–19- and 30–39-year-old respondents (*p* < 0.05). The 60–69 year olds had significantly higher probability of receiving endoscopy compared with 20–59 year olds (*p* < 0.05). Respondents aged 50–59 years had significantly higher probability of undergoing MRI compared with 18–29 year olds (*p* < 0.05) and fluorography compared with 20–29 year olds (*p* < 0.05). Respondents aged 70–89 years had significantly higher probabilities of receiving endoscopy (*p* < 0.05) and X-ray examinations compared with 20–29-year-old respondents (*p* < 0.05).

### 3.3. Morbidity-Related Characteristics

**Figure 3:**
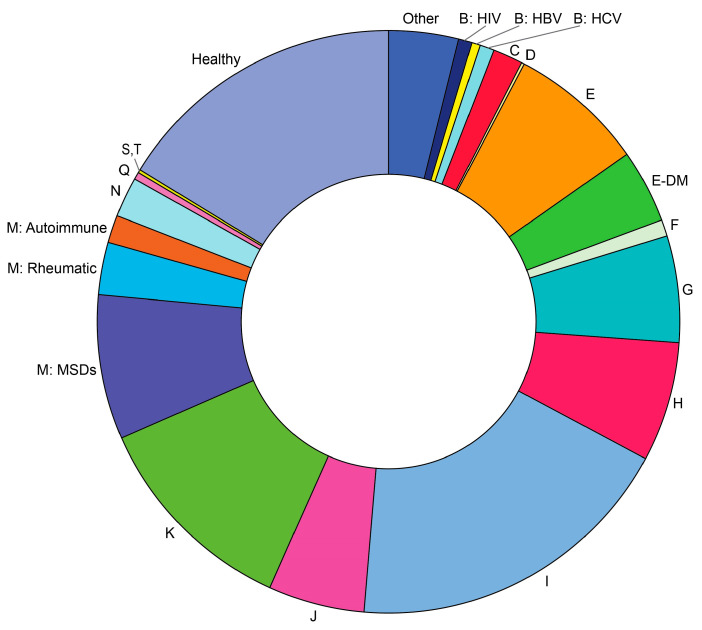
Morbidity structure reported by the respondents of the online survey. B—chronic viral infections, HBV—hepatitis B, HCV—hepatitis C, HIV—human immunodeficiency virus infection. C—oncology disease, D—blood and blood-forming organ diseases and certain disorders involving the immune mechanism, E—endocrine disorders, unspecified, E-DM—diabetes mellitus, F—mental, behavioral, and neurodevelopmental disorders, G—diseases of the nervous system, H—diseases of eye and adnexa, ear, and mastoid process, I—cardiovascular and ischemic diseases, J—diseases of the respiratory system, K—diseases of the digestive system, M—musculoskeletal, rheumatic, and autoimmune disorders, MSDs—musculoskeletal disorders, N—diseases of the genitourinary system, Q—genetic disorders, S,T—injury, poisoning, and certain other consequences of external causes.

**Figure 4:**
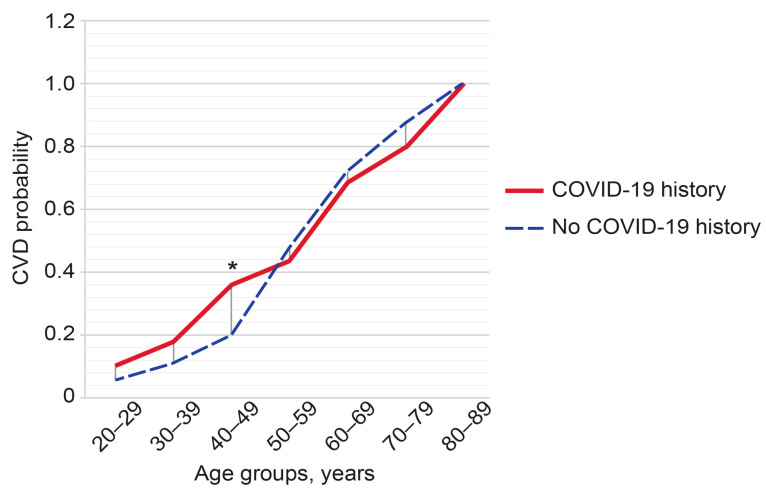
Probabilities of cardiovascular diseases (CVD) in respondents with and without history of COVID-19 depending on age. * *p*-value < 0.05.

Only 27.8% of respondents reported feeling well, while 61.5% of respondents reported slight malady (slightly unwell) and 10.7% felt unwell at the time of survey. As many as 32.8% of respondents reported suffering from cardiovascular disease (CVD); 9.5% of people had chronic respiratory pathology; 28.9% of people in the survey considered themselves free of any chronic diseases. Figure 3 shows a morbidity structure reported by the respondents. Nearly half of respondents (49.7%) had a history of confirmed COVID-19 infection. History of confirmed COVID-19 was associated with a significant increase in CVD morbidity among the respondents aged 40 to 49 years (*p* < 0.05) (Figure 4).

**Figure 5:**
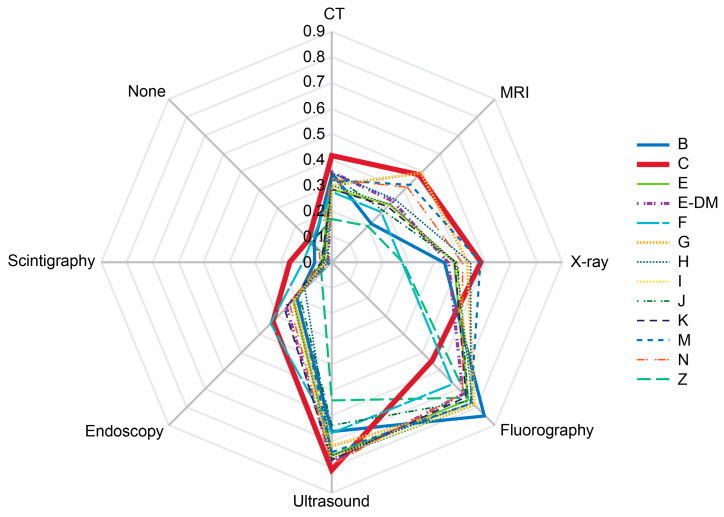
Morbidity-dependent structure of probabilities for the main imaging modalities administered for the past two years prior to the survey. The rates were calculated per person for the past two years prior to the survey. Scale shows calculated probabilities of choosing the corresponding answers. B—chronic viral infections (HBV, HCV, and HIV), C—oncology disease, E—endocrine disorders, unspecified, E-DM—diabetes mellitus, F—mental, behavioral, and neurodevelopmental disorders, G—diseases of the nervous system, H—diseases of eye and adnexa, ear, and mastoid process, I—cardiovascular and ischemic diseases, J—diseases of the respiratory system, K—diseases of the digestive system, M—musculoskeletal, rheumatic, and autoimmune disorders, N—diseases of the genitourinary system, Z—healthy, CT—computed tomography, MRI—magnetic resonance imaging.

Figure 5 shows probabilities of diagnostic imaging administrations within the two years prior to the survey depending on the presence of reported chronic diseases. Respondents with cancer reported a significantly higher rate of scintigraphy (*p* < 0.05) and significantly lower rate of fluorography (*p* < 0.05) compared with respondents of most other groups.

**Figure 6:**
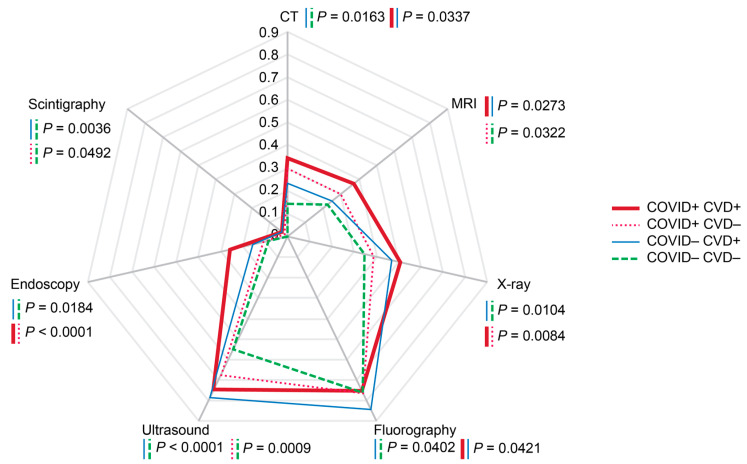
Probabilities of imaging modality administrations depending on history of confirmed COVID-19 in the presence or absence of cardiovascular diseases. The rates were calculated per person for the past two years prior to the survey. Scale shows calculated probabilities of choosing the corresponding answers. CVD—cardiovascular diseases, COVID—history of coronavirus disease 2019.

Respondents with a history of confirmed COVID-19 had significantly higher probabilities of CT (*p* < 0.0001), MRI (*p* < 0.001), ultrasound (*p* < 0.05), and endoscopy (*p* < 0.05) administrations. Figure 6 shows that the presence of CVD was associated with a shift of the diagram towards ultrasound and fluorography among those without a history of COVID-19 (*p* < 0.05). History of COVID-19 in respondents with CVD shifted the diagram towards CT and MRI compared with those who did not have CVD (*p* < 0.05).

The probabilities of receiving fluorography and ultrasound were significantly lower among the respondents who reported to feeling unwell compared with those who felt well or slightly unwell (*p* < 0.05).

### 3.4. Diagnostic Imaging Affordability

Every tenth respondent (10.2%) used to receive diagnostic imaging procedures (MRI, CT, and ultrasound) on privately paid basis, while 29.0% of people in the survey admitted that they were unable to pay for diagnostic imaging procedures. In total, 13.1% of respondents reported unavailability of MRI in their neighborhood. Almost a quarter of respondents (24.0%) reported that MRI was available and covered by compulsory health insurance (CHI), with a waiting time of two to four weeks, while 6.5% reported that insurance-covered MRI was available immediately without waiting. Receiving MRI on a paid basis was associated with a waiting time of two to four weeks in 15.3% of respondents. A total of 41.2% of respondents reported that privately paid MRI was available immediately without any waiting period. At the population level, respondents who reported that they would personally pay for imaging study only in case of emergency (*n* = 656) constituted the vast majority in every diagnostic modality. The top second answer chosen by respondents suggested that they would receive diagnostic study only free of charge, covered by CHI (*n* = 312). Respondents who paid for diagnostic imaging examinations constituted a minority (*n* = 110).

**Figure 7:**
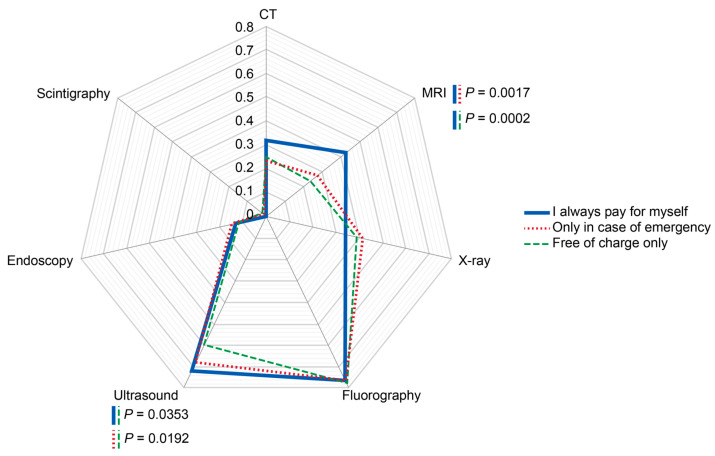
Probabilities of imaging modality administrations depending on respondents’ readiness to cover diagnostic imaging by personal payments. The rates were calculated per person for the past two years prior to the survey. Scale shows calculated probabilities of choosing the corresponding answers. CT—computed tomography, MRI—magnetic resonance imaging.

The rates of administrations for CT, X-ray, fluorography, endoscopy, and scintigraphy did not significantly depend on the ability of respondents to pay for diagnostic imaging (Figure 7). However, there was a significant increase in reported MRI (*p* < 0.001) procedures received by respondents who used to have examinations on a paid basis. Respondents who tended to receive free-of-charge-only diagnostics had the smallest probability of undergoing diagnostic ultrasound (*p* < 0.05).

### 3.5. Professional Status

**Figure 8:**
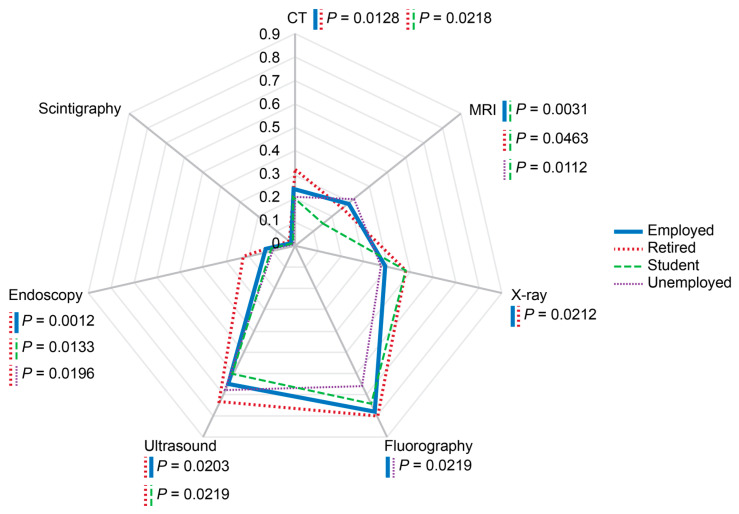
Probabilities of imaging modality administrations depending on professional status of the respondents. The rates were calculated per person for the past two years prior to the survey. Scale shows calculated probabilities of choosing the corresponding answers. CT—computed tomography, MRI—magnetic resonance imaging.

Ultrasound, endoscopy, and CT were most common among retired respondents (*p* < 0.05) (Figure 8). Students reported the lowest rates of MRI administrations (*p* < 0.05). Unemployed individuals had significantly lower probability of receiving fluorography compared with employed participants (*p* < 0.05).

### 3.6. Perceived Superiority of Diagnostic Imaging Modalities

In total, 59.5% of respondents considered CT the most informative method for detecting COVID-19-associated pneumonia. MRI, fluorography, X-ray, and scintigraphy were chosen by 28.4%, 12.5%, 17.5%, and 2.8% of respondents as the best alternatives, respectively.

**Figure 9:**
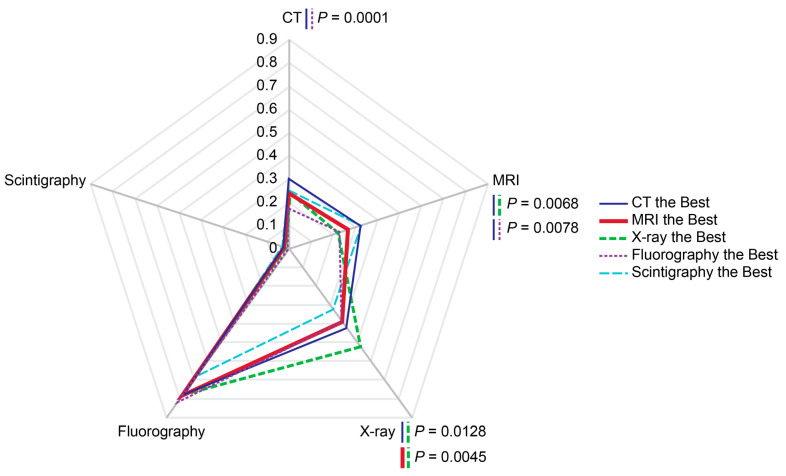
Probabilities of imaging modality administrations depending on perceived superiority of chosen diagnostic modality. The rates were calculated per person for the past two years prior to the survey. Scale shows calculated probabilities of choosing the corresponding answers. CT—computed tomography, MRI—magnetic resonance imaging.

The highest rates of CT, X-ray, fluorography, and scintigraphy tended to occur in the groups of residents who considered these very modalities the best choice for diagnosing COVID-19-assoctiated pneumonia (Figure 9). The only modality that was inconsistent with this pattern was MRI, whose rate was inferior to those of scintigraphy and CT in the group of respondents who, nevertheless, considered MRI the best choice for diagnosing COVID-19-assoctiated pneumonia.

**Figure 10:**
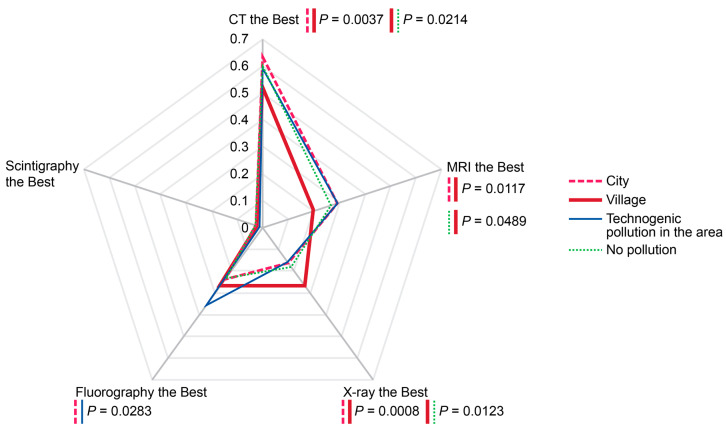
Probabilities of perceived diagnostic superiority of modalities in diagnosing COVID-19-associated pneumonia depending on settlement type and history of past technogenic radioactive events in the area. The rates were calculated per person for the past two years prior to the survey. Scale shows calculated probabilities of choosing the corresponding answers. CT—computed tomography, MRI—magnetic resonance imaging.

Perceived superiority of chosen diagnostic modality depended on settlement type (city or village) and history of technogenic environmentally unfriendly events in the area (Figure 10). Compared with urban residents, the villagers valued diagnostic informativeness of MRI, CT, and fluorography less (*p* < 0.05). In contrast, residents of villages provided a higher rating of X-ray compared with the rating given by respondents residing in the cities. History of technogenic pollution in the area of residence predisposed responders to give a higher appraisal to fluorography (*p* < 0.05) compared with the respondents residing in areas with a more favorable ecological situation.

### 3.7. Local Factors

**Figure 11:**
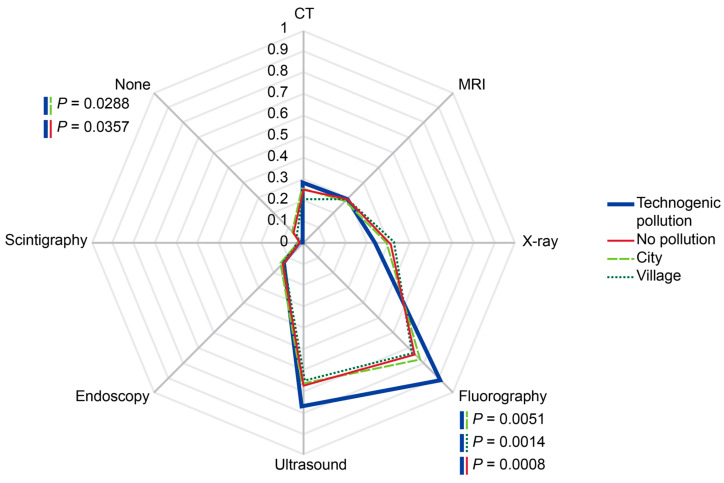
Probabilities of imaging modality administrations depending on local history of technogenic events and settlement type. The rates were calculated per person for the past two years prior to the survey. Scale shows calculated probabilities of choosing the corresponding answers. CT—computed tomography, MRI—magnetic resonance imaging.

Respondents residing in the areas with history of environmentally adverse technogenic events had a significantly higher probability of receiving fluorography compared with respondents living in ecologically favorable areas independently of the type of settlement (*p* < 0.001) (Figure 11).

**Figure 12:**
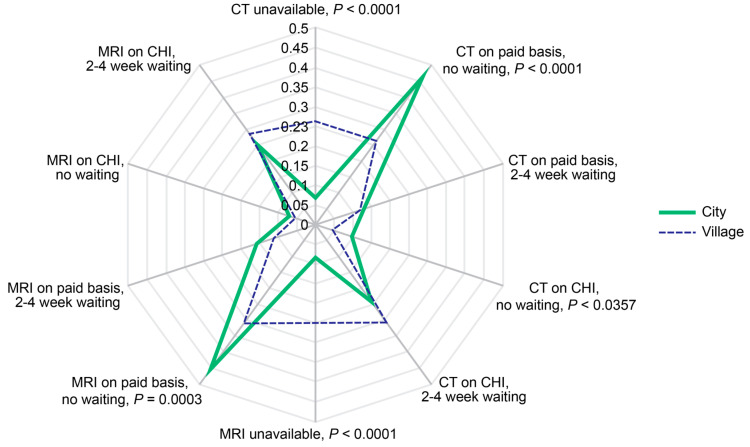
Probabilities of CT and MRI availability as reported by respondents residing in the urban and rural areas, respectively. The rates were calculated per person for the past two years prior to the survey. Scale shows calculated probabilities of choosing the corresponding answers. CHI—compulsory health insurance, CT—computed tomography, MRI—magnetic resonance imaging.

Significantly more respondents from the rural areas considered MRI (*p* < 0.0001) and CT unavailable (*p* < 0.0001). They had a lower probability of receiving CT (*p* < 0.0001) and MRI (*p* < 0.001) on a paid basis without waiting as well as lower probability of receiving prompt CHI-covered CT (*p* < 0.05) (Figure 12).

## 4. Discussion

### 4.1. Present Findings versus Published Research

This paper is the first in a series of articles presenting the results of the online survey-based study registered with ClinicalTrials.gov (NCT06159699). The work uses originally developed websites [33,34] and online survey questionnaire tools. We analyzed the results of the online survey of over a thousand respondents residing in several regions of the Russian Federation. The online survey allowed real-world data to be obtained on population experience with diagnostic imaging at the end of the COVID-19 pandemic and identified the factors influencing probabilities to receive medical imaging. Better understanding of real-world data may contribute to developing remote monitoring approaches for the evaluation of lung disorders [35] and other diseases.

According to the literature, there are many approaches to recruit participants to online surveys, and the methods for improving participation rates in national and regional self-administered web/mail surveys remain under development and investigation [36,37,38]. The effective use of newsletters or emails was found to be the most successful recruitment technique, and the reasons for participating in remote surveys comprise intent to advance research, community protection, and, to a lesser degree, incentives. Altruism is among the primary reasons for contributing to community-based research [38]. The approaches to forming a sample range from sequential mixed-mode designs where alternative non-web modes such as mail and telephone are used to follow up with nonrespondents [36] to employing the services of online survey research firms [39,40] that use different methods, sometimes not fully disclosed. Target-SMS tool, provided by a nationwide telecom company, allowed invitations to be distributed to potential respondents.

A total of 10.7% of our respondents reported to “feel unwell”, and about 30% of them considered themselves free of any chronic diseases. In comparison, 15% of American adults perceived themselves as unhealthy according to data of the Health Information National Trends Survey of the U.S. [41]. Below average (bad and very bad) self-perceived health was reported by 8.6% of Russian respondents in the study focusing on inequalities in perceived health in the Russian Federation, 1994–2012 [42]. Nearly a half of our respondents reported a history of confirmed COVID-19 infection, which exceeds the rate of past COVID-19 infection (16.2%) calculated based on data of the World Health Organization [43] and the Russian Federal State Statistics Service [44]. Considering that many cases, first of all, of mild COVID-19 infection could remain unconfirmed, the vast majority of the surveyed population had a history of COVID-19. Respondents who reported a history of confirmed COVID-19 also reported higher rates of CT, MRI, ultrasound, and endoscopy administrations.

A third of our respondents had CVD despite the relatively young mean age of the people surveyed (~45 years). A significant COVID-19-history-associated increase in CVD morbidity occurred in the 40- to 49-year-old group of respondents, which agrees with observations that incident CVD may be a long-term outcome of COVID-19 infection [2,3,45,46,47,48]. Indeed, non-ischemic myocardial fibrosis, exceeding the prevalence in the normal adult population, was found in every third patient with clinical suspicion of cardiovascular post-acute sequelae of SARS-CoV-2 infection. A possible history of myocarditis may explain the persistent symptoms [49]. History of COVID-19 has been linked to an increased risk of developing aortic aneurysms [50,51]. Alarmingly, aortic strain and aortic distensibility abnormalities in the presence of SARS-CoV-2 and multisystem inflammatory syndrome may occur as early as in childhood [52]. CT shows significant long-lasting pulmonary abnormalities up to two years after suffering from severe COVID-19 infection [53]. Controversy remains regarding the informative value of routine brain MRI protocol for long COVID diagnostics [54]. However, advanced imaging modalities, in particular [18F]FDG PET imaging studies show notable hypometabolism in many regions of the brain in individuals with neurological and psychiatric manifestations of long COVID-19 [55]. COVID-19 history shifted imaging administrations towards CT and MRI in respondents with CVD, which agrees with previous data [2].

COVID-19 pandemics posed new threats to general and cardiac health, and imaging services proved to be valuable in identifying emerging health problems [2,3,47,48]. The global COVID-19 pandemic put unprecedented pressure on the healthcare system worldwide. The first wave of this viral infection was associated with a decline in most areas of healthcare such as planned and screening procedures, including examinations requiring MRI [56,57,58,59]. It is not surprising that a significant drop in the number of diagnostic MRI procedures was observed in our cardiovascular center during the first months of the COVID-19 pandemic, though there was a significant increase in the number of cardiac MRI procedures [2]. The decline was, at least, partially compensated by self-administration of medical imaging. The need for cardiac exams may persist considering the direct (to a lesser degree) and largely indirect (through systemic inflammatory reaction) impact of SARS-CoV-2 on the cardiovascular system. In addition, heart damage may be associated with the side effects of polypharmacy often administered to COVID-19 patients [4,60,61].

Professional status of respondents significantly affected the pattern of administered diagnostic modalities. This association could be partially explained by age difference, for example, between students, retired, employed, and unemployed respondents. Nevertheless, the obtained data demonstrated that there were no large disparities between these population categories, and unemployment status did not prevent people from receiving medical imaging procedures, though unemployment history could create self-perceived barriers to healthcare access [62].

CT is considered the most powerful imaging modality for diagnosing COVID-19-assocated pneumonia [63]. However, less than 60% of our respondents perceived CT as superior to other imaging modalities. The widespread use of CT in COVID-19 may cause adverse biological effects. Low-radiation-dose scans may lower exposure levels but they are significantly inferior in the detection of ground-glass opacity, a hallmark of COVID-19 pneumonia [64]. X-ray and chest fluorography are hardly the methods of choice in cases of COVID-19-associated pneumonia, but MRI may indeed possess high diagnostic value, especially if repeated scans are required [48]. MRI was available to the majority of people in the survey, but it was only the fourth most popular diagnostic imaging method despite its high diagnostic value and well-known safety. A relatively high proportion of people could not access or afford MRI procedure due to residing in remote areas. Privately paid MRI was associated with shorter waiting times and was readily available to roughly every tenth person. Being a safe (in the absence of contraindications) method with high diagnostic value, MRI is still not very affordable, while MRI prescriptions in the private and public sectors are sometimes considered inappropriate [65]. The development and timely updates of appropriate use criteria for MRI would benefit society in case of the next pandemic.

In our study, perceived superiority of diagnostic modalities depended on a settlement type and history of environmentally adverse technogenic events in the area. A less favorable ecological situation predisposed responders to give a higher appraisal to chest fluorography, which was administered to those individuals more often. It remains unclear if there were causal relationships in those associations. Evidence suggests that residing/working in the areas with past technogenic events is associated with increased health-related risks [66]. Our study showed that advanced imaging modalities such as MRI and CT were less available for the respondents residing in rural areas, which agrees with other studies [67].

Considering the history of partial collapse of public healthcare during the COVID-19 pandemic, the population will benefit from adequate self-administration of advanced medical imaging if a similar healthcare crisis reoccurs. In case of a new pandemic, relevant knowledge may navigate people to receive advanced imaging, at least, on a paid basis. Real-world data (RWD) regarding population health conditions and attitudes are essential to generate real-world evidence (RWE), which may be applied to medical diagnostics. Interdisciplinary collaboration would help to translate causal question into causal estimate [68].

### 4.2. Limitations of the Study

A potential limitation of our online survey consists in enriching the study cohort with the respondents who left a digital footprint in the fields of healthcare and pharmacy before and who used the iOS and Android devices. It could lead to underrepresentation of other groups of the diverse Russian population. However, our pilot study of groups who had left or had not left a digital shadow in healthcare for the previous three months gained similar results between these categories of respondents. Moreover, the response rate and survey representativeness could be inferior if invitees did not possess devices enabling them to use the mobile versions of online questionnaire. In addition, invitations, potentially distributed in a blind manner, could not allow the bias of respondents’ cohort underrepresentation to be avoided because acceptance of invitations was affected by respondents’ interest in healthcare and medicine by definition. The Tele2 Russia Target-SMS software used health-related digital shadow to ensure high response to tele-invitations. Measuring potential underrepresentation extent is challenging and requires further research. Another limitation of the study is the relatively small group of respondents residing in the areas with a history of technogenic environmentally adverse events.

### 4.3. Future Directions

This study allowed the gaps in our knowledge to be identified and provided rationale for further research. (1) An in-depth study of the associations between population education and attitudes to medical imaging would contribute to creating new approaches to bolstering pandemic preparedness through educating activities. (2) Population awareness and attitudes towards the emerging machine learning and AI-based and robotic technologies in the context of diagnostic services require further exploration. (3) Awareness and attitudes of healthcare providers, including diagnostic imaging specialists, towards the emerging machine learning and AI-based and robotic technologies should be explored to understand the readiness of the professional community to embrace digital transformation revolutionizing the field of medicine. (4) Further research is needed to understand the role of local factors, including the history of technogenic environmentally adverse events, in shaping population attitudes towards imaging modalities, taking into account health-related risks associated with the exposure to low-dose technogenic radiation [62]. (5) Considering the rapidly changing world at the beginning of sixth wave of innovation, continuous research is required for timely identification of vulnerable population groups potentially disadvantaged in terms of access to the state-of-the-art diagnostic services.

## 5. Conclusions

The present online survey-based observational cross-sectional study allowed the experience, basic knowledge, attitudes, and expectations of unstructured population to be elucidated regarding diagnostic imaging. Respondents with a history of COVID-19 infection constituted nearly a half of the surveyed population and reported higher rates of undergoing CT, MRI, and ultrasound procedures. COVID-19 history was associated with an increased CVD morbidity in younger adults and shifted imaging administrations towards CT and MRI in respondents with CVD. Every tenth respondent received MRI, CT, and ultrasound on a paid basis but almost a third of respondents could not pay for diagnostic procedures, while a significant part of the population (13%) reported unavailable diagnostic imaging services at the place of residence. Professional status significantly affected the patterns of diagnostic modalities, and MRI and CT availability differed between the respondents residing in the urban and rural areas. For some reason, history of technogenic events in the area predisposed responders to overestimate diagnostic value of fluorography in regard to detection of COVID-19-associated pneumonia. In the context of impending pandemics, preventive strategies may require using telecommunication technologies to establish dialogue between healthcare providers, biomedical researchers, and the population to ensure effective management and delivery of diagnostic services when needed. Educational and organizational efforts should focus on promoting relevant imaging modalities among the population and preparing people for upcoming implementation of novel diagnostic approaches based on AI, machine learning, deep neural networks, virtual reality, augmented reality, Tactile Internet, and pervasive computing revolutionizing medical imaging. An increase in affordability and availability of advanced imaging is strategically essential for population health.

## Data Availability

Deidentified individual participant data (text, tables, figures, and appendices), underlying the results of the trial, will be shared with researchers to achieve the aims in the approved proposal. Supporting Information may include Study Protocol, Statistical Analysis Plan (SAP), and Clinical Study Report (CSR). Proposals may be submitted up to 36 months following publication of the results of the trial. After 36 months, the data will be available in the Center’s data warehouse but without investigator support other than deposited metadata. Information regarding submitting proposals and accessing data may be requested from the principal investigator by e-mail sent to corresponding author.

## References

[1] Fernandez Alonso C., Fuentes Ferrer M., Llorens P., Burillo G., Alquézar-Arbé A., Jacob J., Montero-Pérez F.J., Aguiló S., Abad Cuñado V., Amer Al Arud L. (2023). Impact of first wave of COVID-19 pandemic on mortality at emergency department in elderly patients with covid and non-covid diagnoses. Gerontology.

[2] Shelkovnikova T.A., Maksimova A.S., Ryumshina N.I., Mochula O.V., Vaizov V.K., Ussov W.Y., Anfinogenova N.D. (2023). Transformative Effect of COVID-19 Pandemic on Magnetic Resonance Imaging Services in One Tertiary Cardiovascular Center. J. Imaging.

[3] Shelkovnikova T.A., Pushnikova E.Y., Baev A.E., Ryabov V.V., Ussov W.Y. (2022). Magnetic resonance syndromes of myocardial damage in patients after new coronavirus infection (COVID-19)—Two typical clinical cases. Sib. J. Clin. Exp. Med..

[4] Higgins V., Sohaei D., Diamandis E.P., Prassas I. (2021). COVID-19: From an acute to chronic disease? Potential long-term health consequences. Crit. Rev. Clin. Lab. Sci..

[5] Todorovic V. (2022). Mild SARS-CoV-2 infection leaves long-lasting effects on cardiovascular health. Nat. Cardiovasc. Res..

[6] Hinds Z., Lockwood P. (2023). A cross-sectional student survey of the impact of the Covid-19 lockdowns on clinical placement in England. Radiography.

[7] Luchian M.-L., Higny J., Benoit M., Robaye B., Berners Y., Henry J.-P., Colle B., Xhaët O., Blommaert D., Droogmans S. (2023). Unmasking Pandemic Echoes: An In-Depth Review of Long COVID’s Unabated Cardiovascular Consequences beyond 2020. Diagnostics.

[8] Davis H.E., Assaf G.S., McCorkell L., Wei H., Low R.J., Re’em Y., Redfield S., Austin J.P., Akrami A. (2021). Characterizing long COVID in an international cohort: 7 months of symptoms and their impact. EClinicalMedicine.

[9] Xie Y., Xu E., Bowe B., Al-Aly Z. (2022). Long-term cardiovascular outcomes of COVID-19. Nat. Med..

[10] Xie Y., Al-Aly Z. (2022). Risks and burdens of incident diabetes in long COVID: A cohort study. Lancet Diabetes Endocrinol..

[11] Mancini D.M., Brunjes D.L., Lala A., Trivieri M.G., Contreras J.P., Natelson B.H. (2021). Use of Cardiopulmonary Stress Testing for Patients with Unexplained Dyspnea Post-Coronavirus Disease. JACC Heart Fail..

[12] Larsen N.W., Stiles L.E., Shaik R., Schneider L., Muppidi S., Tsui C.T., Geng L.N., Bonilla H., Miglis M.G. (2022). Characterization of autonomic symptom burden in long COVID: A global survey of 2,314 adults. Front. Neurol..

[13] Demko Z.O., Yu T., Mullapudi S.K., Varela Heslin M.G., Dorsey C.A., Payton C.B., Tornheim J.A., Blair P.W., Mehta S.H., Thomas D.L. (2022). Post-acute sequelae of SARS-CoV-2 (PASC) impact quality of life at 6, 12 and 18 months post-infection. medRxiv.

[14] Martínez M.J., Cotton M., Phan M.V.T., Becker K., Espasa M., Leegaard T.M., Lisby G., Schneider U.V., Casals-Pascual C. (2024). Viral epidemic preparedness: A perspective from five clinical microbiology laboratories in Europe. Clin. Microbiol. Infect..

[15] Roe K. (2023). Deadly interactions: Synergistic manipulations of concurrent pathogen infections potentially enabling future pandemics. Drug Discov. Today.

[16] Fragua Á., Jiménez-Martín A., Mateos A. (2023). Complex network analysis techniques for the early detection of the outbreak of pandemics transmitted through air traffic. Sci. Rep..

[17] Haileamlak A. (2022). Pandemics Will be More Frequent. Ethiop. J. Health Sci..

[18] Gupta R. (2021). The need for global access to biomedical innovations during pandemics. Nat. Biotechnol..

[19] Choi Y.K. (2021). Emerging and re-emerging fatal viral diseases. Exp. Mol. Med..

[20] Osadebey M., Andersen H.K., Waaler D., Fossaa K., Martinsen A.C.T., Pedersen M. (2021). Three-stage segmentation of lung region from CT images using deep neural networks. BMC Med. Imaging.

[21] Rajpurkar P., Irvin J., Ball R.L., Zhu K., Yang B., Mehta H., Duan T., Ding D., Bagul A., Langlotz C.P. (2018). Deep learning for chest radiograph diagnosis: A retrospective comparison of the CheXNeXt algorithm to practicing radiologists. PLoS Med..

[22] Kwon S., Joshi A.D., Lo C.H., Drew D.A., Nguyen L.H., Guo C.G., Ma W., Mehta R.S., Shebl F.M., Warner E.T. (2021). Association of social distancing and face mask use with risk of COVID-19. Nat. Commun..

[23] Block P., Hoffman M., Raabe I.J., Dowd J.B., Rahal C., Kashyap R., Mills M.C. (2020). Social network-based distancing strategies to flatten the COVID-19 curve in a post-lockdown world. Nat. Hum. Behav..

[24] Memos V.A., Minopoulos G., Stergiou K.D., Psannis K.E. (2021). Internet-of-things-enabled infrastructure against infectious diseases. IEEE Internet Things Mag..

[25] Minopoulos G.M., Memos V.A., Stergiou K.D., Stergiou C.L., Psannis K.E. (2023). A medical image visualization technique assisted with AI-based haptic feedback for robotic surgery and healthcare. Appl. Sci..

[26] Arul Raj A.M., Sugumar R. Enhancing COVID-19 diagnosis with automated reporting using preprocessed chest X-ray image analysis based on CNN. Proceedings of the 2023 2nd International Conference on Applied Artificial Intelligence and Computing (ICAAIC).

[27] Ding X., Zhou Q., Liu Z., Hammad Kowah J.A., Wang L., Huang X., Liu X. (2024). A Novel Approach to the Technique of Lung Region Segmentation Based on a Deep Learning Model to Diagnose COVID-19 X-ray Images. Curr. Med. Imaging.

[28] Kundu A., Mishra C., Bilgaiyan S. COVID-SEGNET: Diagnosis of COVID-19 cases on radiological images using mask R-CNN. Proceedings of the 2021 Seventh International Conference on Bio Signals, Images, and Instrumentation (ICBSII).

[29] Karim M.R., Döhmen T., Cochez M., Beyan O., Rebholz-Schuhmann D., Decker S. DeepCOVIDExplainer: Explainable COVID-19 diagnosis from chest X-ray images. Proceedings of the 2020 IEEE International Conference on Bioinformatics and Biomedicine (BIBM).

[30] Ghafoori M., Hamidi M., Modegh R.G., Aziz-Ahari A., Heydari N., Tavafizadeh Z., Pournik O., Emdadi S., Samimi S., Mohseni A. (2023). Predicting survival of Iranian COVID-19 patients infected by various variants including omicron from CT Scan images and clinical data using deep neural networks. Heliyon.

[31] Di Gessa G., Price D. (2021). Changes in health and social well-being in the COVID-19 clinically vulnerable older English population during the pandemic. J. Epidemiol. Community Health.

[32] Bann D., Villadsen A., Maddock J., Hughes A., Ploubidis G.B., Silverwood R., Patalay P. (2021). Changes in the behavioural determinants of health during the COVID-19 pandemic: Gender, socioeconomic and ethnic inequalities in five British cohort studies. J. Epidemiol. Community Health.

[33] Questionnaire. Online Survey-Based Clinical Study: Risk Factors and New Challenges for the Population on the Path to Achieving Active Aging. https://zdorov.expert/1-1.

[34] Online Survey-Based Clinical Study: Risk Factors and New Challenges for the Population on the Path to Achieving Active Aging. https://zdorov.tpu.ru/forms.

[35] Ranjan Y., Althobiani M., Jacob J., Orini M., Dobson R.J., Porter J., Hurst J., Folarin A.A. (2021). Remote Assessment of Lung Disease and Impact on Physical and Mental Health (RALPMH): Protocol for a Prospective Observational Study. JMIR Res. Protoc..

[36] West B.T., Zhang S., Wagner J., Gatward R., Saw H.W., Axinn W.G. (2023). Methods for improving participation rates in national self-administered web/mail surveys: Evidence from the United States. PLoS ONE.

[37] Edwards P.J., Roberts I., Clarke M.J., DiGuiseppi C., Woolf B., Perkins C. (2023). Methods to increase response to postal and electronic questionnaires. Cochrane Database Syst. Rev..

[38] Schilling J., Klein D., Bartholmae M.M., Shokouhi S., Toepp A.J., Roess A.A., Sill J.M., Karpov M.V., Maney K., Brown K.P. (2023). A Digital Health Initiative (COVIDsmart) for Remote Data Collection and Study of COVID-19’s Impact on the State of Virginia: Prospective Cohort Study. JMIR Form. Res..

[39] Lakhoo K., Almario C.V., Khalil C., Spiegel B.M.R. (2021). Prevalence and Characteristics of Abdominal Pain in the United States. Clin. Gastroenterol. Hepatol..

[40] Lee J.J., Poon C.Y., O’Connor S., Wong J.Y.H., Kwok J.Y.Y., Choi E.P.H., Tsang W.N., Wang M.P. (2023). Associations of eHealth literacy and knowledge with preventive behaviours and psychological distress during the COVID-19 pandemic: A population-based online survey. BMJ Open.

[41] AlDukhail S., Bahdila D. (2022). Self-perception of health and physical activity among adults before and amidst the COVID-19 pandemic: United States, 2019–2020. Prev. Med..

[42] Paul P., Valtonen H. (2016). Inequalities in perceived health in the Russian Federation, 1994–2012. BMC Public Health.

[43] World Health Organization Number of COVID-19 Cases Reported to WHO (Cumulative Total): Russian Federation. https://data.who.int/dashboards/covid19/cases?m49=643&n=c.

[44] Federal State Statistics Service (17 March 2023) Estimation of the Resident Population as of 1 January 2023 and on Average for 2022 and the Components of Its Change (Taking into Account the Results of the All-Russian Population Census of 2020). https://rosstat.gov.ru/compendium/document/13282.

[45] Koyama A.K., Imperatore G., Rolka D.B., Lundeen E., Rutkowski R.E., Jackson S.L., He S., Kuklina E.V., Park S., Pavkov M.E. (2023). Risk of Cardiovascular Disease After COVID-19 Diagnosis Among Adults with and without Diabetes. J. Am. Heart Assoc..

[46] Ussov W.Y., Ignatenko G.A., Nudnov N.V., Bergen T.A., Gulyaev V.M., Pervak M.B., Yaroshevsky S.P., Dubovaya A.V., Karmazanovsky G.G. Comprehensive MRI of the Chest and Brain in the Diagnosis of Injury to Thoracic Organs, Myocardium, and Brain in COVID-19. The University Clinic 2021. Suppl. I, pp. 144–145. https://dnmu.ru/wp-content/uploads/2021/02/materConfCovid_010221.pdf.

[47] Ussov W.Y., Nudnov N.V., Ignatenko G.A., Fisenko A.Y., Gulyaev V.M., Maritskii S.V., Kalyuzhin V.V., Lukyanenok P.I. (2020). Evaluation of lung damage in pneumonia, from chest magnetic resonance imaging, in primary diagnosis and in the follow-up of treatment. Med. Vis..

[48] Ussov W.Y., Nudnov N.V., Ignatenko G.A., Gulyaev V.M., Pervak M.B., Shelkovnikova T.A., Dubovaya A.V., Bergen T.A. (2020). Primary and prospective imaging of the chest using magnetic resonance imaging in patients with viral lung damage in COVID-19. Med. Vis..

[49] Halfmann M.C., Luetkens J.A., Langenbach I.L., Kravchenko D., Wenzel P., Emrich T., Isaak A. (2023). Cardiac MRI Findings in Patients Clinically Referred for Evaluation of Post-Acute Sequelae of SARS-CoV-2 Infection. Diagnostics.

[50] Mohseni A., Di Girolamo A., Cangiano R., Ascione M., di Marzo L., Mansour W. (2024). High-Grade Infection after Branched Endovascular Aortic Repair in Patient with Recent COVID-19 Hospitalization. Diagnostics.

[51] Anfinogenova N.D., Sinitsyn V.E., Kozlov B.N., Panfilov D.S., Popov S.V., Vrublevsky A.V., Chernyavsky A., Bergen T., Khovrin V.V., Ussov W.Y. (2022). Existing and Emerging Approaches to Risk Assessment in Patients with Ascending Thoracic Aortic Dilatation. J. Imaging.

[52] Ostberg N.P., Zafar M.A., Ziganshin B.A., Elefteriades J.A. (2020). The Genetics of Thoracic Aortic Aneurysms and Dissection: A Clinical Perspective. Biomolecules.

[53] Babar M., Jamil H., Mehta N., Moutwakil A., Duong T.Q. (2024). Short- and Long-Term Chest-CT Findings after Recovery from COVID-19: A Systematic Review and Meta-Analysis. Diagnostics.

[54] Vasilev Y., Blokhin I., Khoruzhaya A., Kodenko M., Kolyshenkov V., Nanova O., Shumskaya Y., Omelyanskaya O., Vladzymyrskyy A., Reshetnikov R. (2023). Routine Brain MRI Findings on the Long-Term Effects of COVID-19: A Scoping Review. Diagnostics.

[55] Hameed R., Bahadur A.R., Singh S.B., Sher J., Todua M., Moradi L., Bastakoti S., Arslan M., Ajmal H., Lee G.Y. (2023). Neurological and Psychiatric Manifestations of Long COVID-19 and Their [18F]FDG PET Findings: A Review. Diagnostics.

[56] de Pelsemaeker M.C., Guiot Y., Vanderveken J., Galant C., Van Bockstal M.R. (2021). The Impact of the COVID-19 Pandemic and the Associated Belgian Governmental Measures on Cancer Screening, Surgical Pathology and Cytopathology. Pathobiology.

[57] Keizman E., Ram E., Kachel E., Sternik L., Raanani E. (2020). The impact of COVID-19 pandemic on cardiac surgery in Israel. J. Cardiothorac. Surg..

[58] Saban M., Reznik A., Shachar T., Wilf-Miron R., Sivan-Hoffmann R. (2021). The effect of the COVID-19 pandemic on ED referrals and care for stroke patients: A four-year comparative study. J. Crit. Care.

[59] Hartnett K.P., Kite-Powell A., DeVies J., Coletta M.A., Boehmer T.K., Adjemian J., Gundlapalli A.V., National Syndromic Surveillance Program Community of Practice (2020). Impact of the COVID-19 Pandemic on Emergency Department Visits—United States, January 1, 2019—May 30, 2020. MMWR Morb. Mortal. Wkly. Rep..

[60] Anfinogenova N.D., Trubacheva I.A., Popov S.V., Efimova E.V., Ussov W.Y. (2021). Trends and concerns of potentially inappropriate medication use in patients with cardiovascular diseases. Expert Opin. Drug Saf..

[61] Anfinogenova N.D., Novikova O.M., Trubacheva I.A., Efimova E.V., Chesalov N.P., Ussov W.Y., Maksimova A.S., Shelkovnikova T.A., Ryumshina N.I., Stepanov V.A. (2023). Prescribed Versus Taken Polypharmacy and Drug–Drug Interactions in Older Cardiovascular Patients during the COVID-19 Pandemic: Observational Cross-Sectional Analytical Study. J. Clin. Med..

[62] Hoven H., Backhaus I., Gerő K., Kawachi I. (2023). Characteristics of employment history and self-perceived barriers to healthcare access. Eur. J. Public Health.

[63] Xu X., Yu C., Qu J., Zhang L., Jiang S., Huang D., Chen B., Zhang Z., Guan W., Ling Z. (2020). Imaging and clinical features of patients with 2019 novel coronavirus SARS-CoV-2. Eur. J. Nucl. Med. Mol. Imaging.

[64] Karakaş H.M., Yıldırım G., Çiçek E.D. (2021). The reliability of low-dose chest CT for the initial imaging of COVID-19: Comparison of structured findings, categorical diagnoses and dose levels. Diagn. Interv. Radiol..

[65] Jahanmehr N., Bigdeli A.S., Salari H., Mokarami H., KhodaKarim S., Damiri S. (2019). Analyzing inappropriate magnetic resonance imaging (MRI) prescriptions and resulting economic burden on patients suffering from back pain. Int. J. Health Plann. Manag..

[66] Karpov A.B., Takhauov R.M., Zerenkov A.G., Semenova Y.V., Bogdanov I.M., Kazantceva S.B., Blinov A.P., Kalinkin D.E., Gorina G.V., Litvinova O.V. (2021). Descriptive characteristics of occupational exposures and medical follow-up in the cohort of workers of the Siberian Group of Chemical Enterprises in Seversk, Russia. Int. J. Radiat. Biol..

[67] Burdorf B.T. (2021). Comparing magnetic resonance imaging and computed tomography machine accessibility among urban and rural county hospitals. J. Public Health Res..

[68] Dang L.E., Gruber S., Lee H., Dahabreh I.J., Stuart E.A., Williamson B.D., Wyss R., Díaz I., Ghosh D., Kıcıman E. (2023). A causal roadmap for generating high-quality real-world evidence. J. Clin. Transl. Sci..

